# 

**Published:** 1897-07

**Authors:** 


ESTABLISHED 1855. SUBSCRIPTION, $2.00.

### ATLANTA MEDICAL AND SURGICAL JOURNAL.

VOLUME XIV NEW SERIES. ATLANTA, GA., JULY, 1897. No. 5.

